# Design rationale of thermally responsive microgel particle films that reversibly absorb large amounts of CO_2_: fine tuning the p*K*_a_ of ammonium ions in the particles†
†Electronic supplementary information (ESI) available. See DOI: 10.1039/c5sc01978h


**DOI:** 10.1039/c5sc01978h

**Published:** 2015-07-28

**Authors:** Mengchen Yue, Yu Hoshino, Yoshiko Miura

**Affiliations:** a Department of Chemical Engineering , Kyushu University , 744 Motooka, Nishi-ku , Fukuoka 819-0395 , Japan . Email: yhoshino@chem-eng.kyushu-u.ac.jp

## Abstract

Herein we revealed the design rationale of thermally responsive gel particle (GP) films that reversibly capture and release large amounts of CO_2_ over a narrow temperature range (30–75 °C). The p*K*_a_ value of ammonium ions in the GPs at both the CO_2_ capture temperature (30 °C) and release temperature (75 °C) is found to be the primary factor responsible for the stoichiometry of reversible CO_2_ capture by the amines in the GP films. The p*K*_a_ values can be tuned by the properties of GPs such as volume phase transition temperature (VPTT), size, swelling ratio, and the imprinted microenvironment surrounding the amines. The optimal GP obtained according to the design rationale showed high capture capacity (68 mL CO_2_ per g dry GPs, 3.0 mmol CO_2_ per g dry GPs), although the regeneration temperature was as low as 75 °C. We anticipate that GP films that reversibly capture other acidic and basic gases in large amounts can also be achieved by the p*K*_a_ tuning procedures.

## Abstract 2

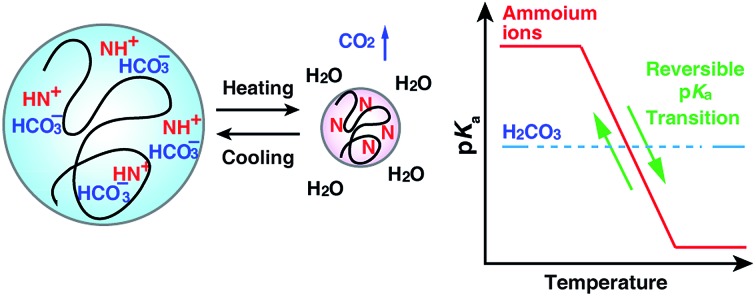
Fine-tuning of p*K*_a_ value of ammonium ions at both CO_2_ capture and release temperature is found to be crucial for the design of the thermally responsive gel particle films that reversibly capture large amounts of CO_2_.

## Introduction

The accumulation of CO_2_ in the atmosphere due to burning of fossil fuels is considered the main cause of global climate change.^1^ Meanwhile, CO_2_ is expected to be a carbon source for the production of liquid fuels, such as methanol and dimethyl ether, through the use of regenerative energy.^2^ Thus, the development of energy-efficient CO_2_ separation and recovery processes for point sources, such as fossil fuel power plants, is essential not only for minimizing climate change but also for the future use of carbon-based energy.

Conventional processes that recover CO_2_ from the high humidity exhaust gases of power plants use aqueous solutions of ethanolamine as a CO_2_ absorbent. CO_2_ in the exhaust gases is selectively captured by the absorbent at a low temperature (∼40 °C) and then recovered by heating the solution, typically above 140 °C.^3^–^5^ Although the amine solution exhibits a high CO_2_ absorption capacity, the high-energy consumption of the heating process limits its use in the environmentally friendly process.^4^–^8^

Porous solid adsorbents that can be regenerated under relatively mild conditions, such as metal–organic frameworks (MOFs),^9^,^10^ zeolites,^9^,^10^ and silica with physically or chemically supported amine polymers,^9^–^13^ have recently been developed as alternatives to the aqueous amine solution. However, solid CO_2_ sorbents are rarely capable of efficiently capturing CO_2_ from highly humid gases because the adsorption of water molecules competes with that of CO_2_ on the pore surfaces. Furthermore, the capillaries and pores of the sorbents are easily blocked by the liquefied water, preventing CO_2_ from diffusing into the pores.^9^–^12^ Thus, the development of sorbents that absorb/adsorb large amount of CO_2_, even in humid environments, and desorb CO_2_ at low temperatures (<100 °C) is of great importance.

It has been reported that poly-*N*-isopropylacrylamide (pNIPAm) undergoes a reversible volume phase transition from a hydrophilic swollen state to a hydrophobic collapsed state at temperatures below and above, respectively, the volume phase transition temperature (VPTT) of ∼32 °C.^14^ The phase transition is induced by the entropy-driven dissociation of water from pNIPAm chains after heating above the VPTT.^14^ The temperature responsive pNIPAm-based functional hydrogels have been widely used as materials to reversibly capture targets, such as dyes,^15^,^16^ drugs,^17^,^18^ peptides,^19^ proteins,^20^,^21^ nucleotides,^22^ cells,^23^ and protons,^24^ in aqueous media *via* the volume phase transition. The multipoint interactions are reversibly switched on/off by varying the volume density of hydrophobic functional groups,^17^,^25^,^26^ the number of charged functional groups,^22^,^27^ and/or the rigidity of the polymer chains^28^ at temperatures above and below the VPTT.

Recently, we reported that hydrogel films composed of temperature-responsive microgel particles (GPs) consisting of NIPAm and *N*-[3-(dimethylamino)propyl]methacrylamide (DMAPM) reversibly absorbed and released CO_2_*via* a volume phase transition during cooling (30 °C) and heating (75 °C) cycles, respectively.^29^ Below the VPTT, amines in the swollen GPs were capable of forming ion pairs with absorbed bicarbonate ions. Above the VPTT, shrinkage of the GPs triggered CO_2_ desorption.^27^ The GP films showed faster CO_2_ capture and release rate^29^ than the conventional bulk hydrogel films due to the fast thermal responsibility of the GPs.^30^

However, guidelines to design GP films that reversibly absorb CO_2_ with high capacity and stoichiometry have not been revealed.

In the case of reversible CO_2_ absorption by the blood of animals, the p*K*_a_ value of the Brønsted acid and base in the hemoglobin plays a crucial role for the control of CO_2_ solution equilibrium: The p*K*_a_ value of the ammonium and imidazolium cations decreases due to the drastic conformational change of the hemoglobin caused by oxygen binding to the heme. The shift of the p*K*_a_ triggers the release of H^+^ into the blood, leading to the release of CO_2_ efficiently from lungs (Bohr effect).^31^,^32^

Inspired by the Bohr effect of hemoglobin, we hypothesized that the p*K*_a_ value of the protonated amines (ammonium ions) within GPs (p*K*_a_ value of GPs) at the CO_2_ capture temperature (30 °C) and release temperature (75 °C) would contribute to the reversible capture efficiency of CO_2_.

In this study, in order to clarify the design rationale, we prepared a series of GPs with a variety of compositions using different polymerization conditions. The effects of the physical and chemical properties of GPs, such as VPTT, size, swelling ratio, on p*K*_a_ values and the reversible CO_2_ capture stoichiometry against amines were systematically investigated. p*K*_a_ values of the ammonium ions in the GPs were also tuned by the “microenvironment-imprinting” strategy as we described in the recent report.^24^ The reversible CO_2_ capture capacity was maximized based on the design rational revealed in this study. Humidified gas (60 °C) consisting of 10% CO_2_ and 90% N_2_, which is comparable to the post-combustion gas of fire power plants after wet desulfurization process, was used as feed gas.^6^,^33^ The CO_2_ was captured at 30 °C and released at 75 °C under the same atmosphere (10% CO_2_, 90% N_2_, 60 °C water moisture).

## Experimental

### Preparation of GPs

**Scheme 1:**
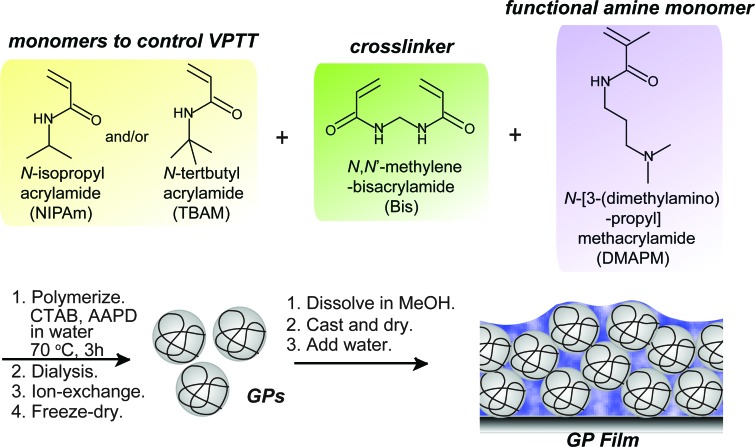
Synthesis of GPs and preparation of GP films.

A series of GPs containing NIPAm, a functional tertiary amine monomer DMPAM, and a crosslinker *N*,*N*′-methylenebisacrylamide (Bis) were synthesized by precipitation polymerization as reported (Scheme 1).^29^ The amount of Bis was varied to prepare GPs with different degrees of crosslinking and swelling ratios. GPs with the same composition but different p*K*_a_ values were synthesized *via* a “microenvironment-imprinting” strategy by adding HCl or NaOH into the monomer solution.^24^ Larger GPs were obtained by decreasing the concentration of surfactant, while smaller GPs were prepared from solutions with a lower total monomer concentration. To decrease the VPTT of GPs, a more hydrophobic monomer, *N-tert*-butyl acrylamide (TBAm), was incorporated into the GPs. Details of the polymerization conditions of the GPs are summarized in Table 1. Polymerization process is described in ESI.†

**Table 1:** Polymerization conditions of the GPs

GPs	Feed ratio				Polymerization media	CTAB (mM)	AAPD (mM)	Concentration (mM)
	NIPAm (mol%)	DMAPM (mol%)	Bis (mol%)	TBAM (mol%)				
**D5B2**	93	5	2	0	Water	2.0	2.5	312
**D5B0**	95	5	0	0	Water	2.0	2.5	312
**D5B5**	90	5	5	0	Water	2.0	2.5	312
**D5B10**	85	5	10	0	Water	2.0	2.5	312
**D5B2T40**	53	5	2	40	Water	2.0	2.5	312
**D5B10-1/4HCl**	85	5	10	0	1/4 eq. HCl	2.0	2.5	312
**D5B2-1/2HCl**	93	5	2	0	1/2 eq. HCl	2.0	2.5	312
**D5B2-1/1HCl**	93	5	2	0	1/1 eq. HCl	2.0	2.5	312
**D5B2-1/2NaOH**	93	5	2	0	1/2 eq. NaOH	2.0	2.5	312
**D5B2-L**	93	5	2	0	Water	0.16	2.5	312
**D5B2-S**	93	5	2	0	Water	2.0	0.625	78
**D5B2-1/2NaOH-S**	93	5	2	0	1/2 eq. NaOH	2.0	0.625	78
**D30B2**	68	30	2	0	Water	2.0	2.5	312
**D30B2T40**	28	30	2	40	Water	2.0	2.5	312
**D55B2**	43	55	2	0	Water	2.0	2.5	312
**D55B2T43**	0	55	2	43	Water	2.0	2.5	312

### Quantification of hydrodynamic diameters, VPTTs, and swelling ratios of GPs

The hydrodynamic diameters and VPTT of GPs were measured by dynamic light scattering (DLS) as described.^27^,^29^ The swelling ratio is defined as the ratio of the diameters at 30 °C (*D*_30_) and 75 °C (*D*_75_).

### Quantification of amine content and p*K*_a_ of ammonium ions in GPs

The amine content and p*K*_a_ of amines in GPs was quantified by acid–base titration under N_2_ purging as described.^27^,^29^

### Measurement of p*K*_a_ shift curve during heating process

Since the apparent p*K*_a_ can be approximated as the pH value at the half-neutralization point, the temperature dependent p*K*_a_ shift of the GP solution was measured with the pH probe as follows. The half-neutralized GP solution was prepared by adding 0.5 eq. of HCl to the GP solution. Then, under a N_2_ atmosphere with stirring the pH value and temperature of the solution were recorded by the pH meter every 3 s during the heating process.

### Preparation of GP films

The lyophilized GPs were dissolved in methanol. The hydrogel films were then prepared by casting the methanol solutions containing 120 mg of GPs on the inner bottom surface of a stainless steel container with a surface area of 120 cm^2^. After completely evaporating the methanol, 4 mL of water was added per gram of GPs.

### Measurement of CO_2_ absorption capacity of GP films

The CO_2_ absorption capacities of the GP films are quantified as illustrated in Scheme S1.† 10% CO_2_ (90% N_2_, 10 mL min^–1^) gas was passed through 60 °C water to generate saturated water vapor. The resulting 60 °C water–saturated gas mixture was channeled into a stainless steel container with the GP film on the inner surface, and then to a gas chromatograph after condensing the water moisture at 5 °C.

More detailed information about materials and experimental process are shown in ESI.†

## Results and discussion

### Effect of p*K*_a_ values of GPs on the reversible CO_2_ capture stoichiometry of GP films

The dynamic and reversible transition of the p*K*_a_ values of Brønsted acid and base groups in proteins plays an important role in their functions, such as molecule/ion transfer, enzymatic reactions, and molecular recognition.^32^,^34^ The p*K*_a_ values are significantly influenced by the microenvironment around the functional groups, such as hydrophobicity, hydrogen bonding, and distance to the neighboring charges.^32^,^34^–^38^ Thus, the p*K*_a_ value can be further dynamically shifted by the conformational change of polypeptides around the functional groups, which induces a microenvironment change.

In the case of CO_2_ transfer through the bloodstream of animals, the p*K*_a_ value of the ammonium and imidazolium cations in/on the hemoglobin decreases due to the drastic conformational change of the polypeptides caused by oxygen binding to the heme. The shift of the p*K*_a_ value triggers the release of H^+^ into the blood, lowers the pH of the blood, and drives the CO_2_ efficiently from the lungs (Bohr effect).^32^,^38^

Inspired by the function of hemoglobin, we expected that the p*K*_a_ value of the protonated amines (ammonium ions) within GPs (p*K*_a_ value of GPs) at the CO_2_ capture temperature (30 °C) and release temperature (75 °C) would contribute to the reversible capture efficiency of CO_2_.

To clarify the effect of the p*K*_a_ value of GPs on the reversible CO_2_ capture stoichiometry of GP films in the temperature swing process from 30 °C to 75 °C, a series of GPs with the same composition but different p*K*_a_ values were prepared by the “microenvironment-imprinting” strategy.^24^,^39^ Imprinting is a method to create polymers with specific microstructures that show strong affinity to target molecules and ions by crosslinking the polymer networks in the presence of targets.^40^,^41^ It has been reported that pNIPAm-based acrylic acids (AAc) containing GPs with different p*K*_a_ values can be prepared by the “proton-imprinted” strategy.^24^ The GPs polymerized at a pH below the p*K*_a_ of AAc showed a much higher p*K*_a_ value at the collapsed state than those prepared at a high pH because stronger proton-affinity sites were incorporated into the relatively hydrophobic microenvironment around the protonated AAc. Note that the GPs were polymerized at the collapsed state in the temperature above the VPTT.

We anticipated that amine-containing GPs with different p*K*_a_ values could also be prepared *via* the “microenvironment-imprinting” strategy. Thus, the GPs were polymerized in the presence of acid (1 or 1/2 eq. of HCl against the amine monomer DMAPM) or base (1/2 eq. of NaOH). All GPs were polymerized in the aqueous monomer solution consisting of 5 mol% DMAPM, 2 mol% Bis and 93 mol% NIPAm at the temperature above the VPTT of the growing GPs (70 °C).

The VPTTs of the GPs were determined by monitoring the scattering intensity of the GP solutions during the heating process (Fig. S1a in ESI†). Despite the different polymerization conditions, all four GPs, **D5B2-1/1HCl** (1 eq. HCl), **D5B2-1/2HCl** (1/2 eq. HCl), **D5B2-1/2NaOH**, (1 eq. NaOH), and **D5B2** (without HCl or NaOH), show similar VPTTs at about 38 °C. Similar swelling ratios in the range of 2.3–2.7 are also observed (Fig. S1b in ESI†), indicating the VPTTs and swelling ratios of GPs are independent of the polymerization conditions.

**Fig. 1:**
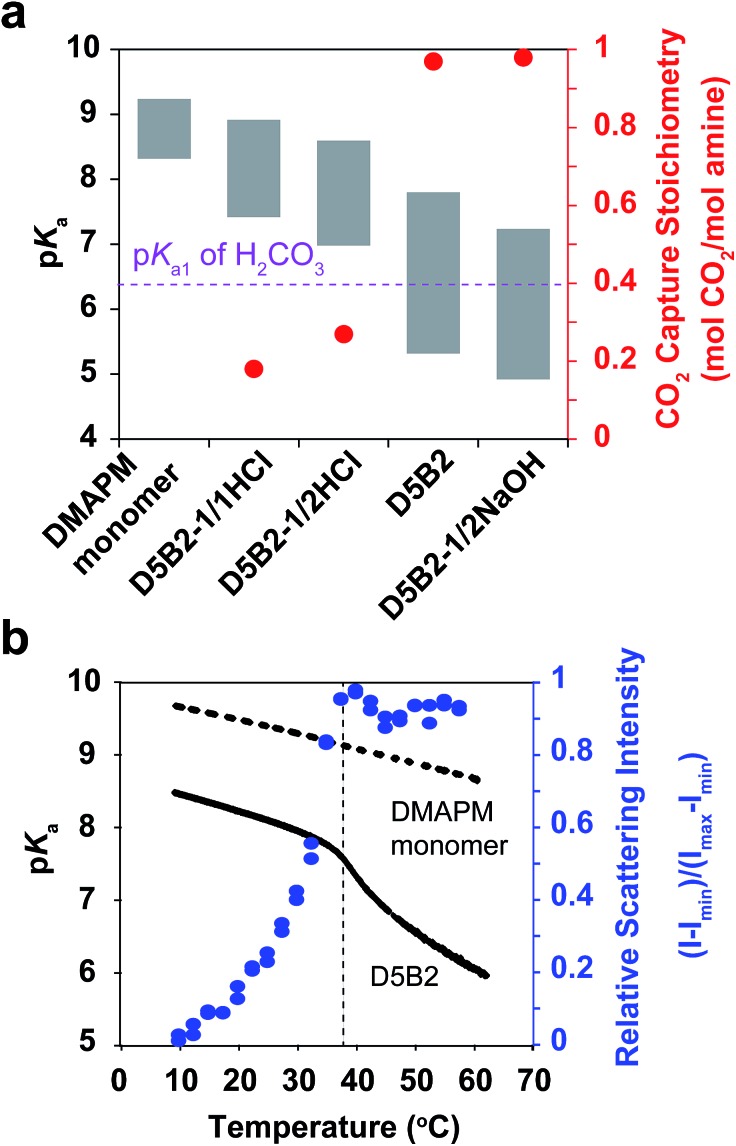
(a) p*K*_a_ values (left *Y*-axis) of **D5B2-1/1HCl**, **D5B2-1/2HCl**, **D5B2**, **D5B2-1/2NaOH** and the monomer DMAPM at 75 °C (bottom of bar) and 30 °C (top of bar), and the reversible CO_2_ capture stoichiometries (right *Y* axis, red dot) of the GP films in the temperature range 30–75 °C. (b) p*K*_a_ shift (left *Y*-axis) of **D5B2** (black solid line) and the monomer DMAPM (black dashed line) during the heating process, and the temperature dependent relative scattering intensity (right *Y*-axis, blue dot) of **D5B2**.

The apparent p*K*_a_ values of the GPs at 30 °C and 75 °C were determined by acid–base titration of the GP solutions and are plotted as the top and bottom, respectively, of the gray bars in Fig. 1a.^27^ All GPs show lower p*K*_a_ values than the amine monomer DMAPM, at both temperatures, indicating the relatively low dielectric constant and high steric hindrance around the amine groups in the GPs, as well as the high electrostatic repulsion between neighboring charges.^42^,^43^ Moreover, in accordance with our expectation, the p*K*_a_ values of **D5B2-1/1HCl** and **D5B2-1/2HCl** at 75 °C (7.4 and 7.0, respectively) were significantly higher than those of **D5B2** and **D5B2-1/2NaOH** (5.3 and 4.9, respectively).

The large difference in p*K*_a_ values can be explained by the “microenvironment-imprinting” effect. When the GPs are polymerized in a proton-rich solution (in the presence of HCl), the protonated DMAPM is imprinted in the polar and hydrophilic microenvironment of the GPs (**D5B2-1/1HCl** and **D5B2-1/2HCl**), resulting in higher p*K*_a_ values than those of the GPs polymerized in the proton-poor solution (**D5B2** and **D5B2-1/2NaOH**). Although there is variation in the GP diameters (Fig. S1b in ESI†), the large difference in the p*K*_a_ values (>2) at 75 °C cannot be a result of the size differences because the smallest GP, **D5B2** (74 nm), and the largest GP, **D5B2-1/2NaOH** (136 nm), show a p*K*_a_ difference of only 0.4 (5.3–4.9).

Fig. 1a also shows that the p*K*_a_ difference between 30 °C and 75 °C (Δp*K*_a_) of all GPs (1.5–2.5) is larger than that of the monomer (0.9). For better understanding of the temperature dependent p*K*_a_ difference, the apparent p*K*_a_ values of **D5B2** and the monomer DMAPM, were recorded during the heating process. Fig. 1b shows that a sharp p*K*_a_ transition of **D5B2** occurs at a temperature around its VPTT, and a steep p*K*_a_ decrease continues over a wide temperature range above the VPTT. However, this sharp p*K*_a_ transition is not observed in the case of the monomer, indicating that the sharp p*K*_a_ decrease was induced by a volume phase transition of GPs, during which the increased hydrophobicity and steric hindrance, and the decreased dielectric constant at high temperature make the amine groups more difficult to be protonated.^42^

In Fig. 1a, the Δp*K*_a_ values between 75 °C and 30 °C of the GPs polymerized with HCl, **D5B2-1/1HCl** (1.5) and **D5B2-1/2HCl** (1.6), are significantly lower than those of **D5B2** (2.5) and **D5B2-1/2NaOH** (2.3), although the swelling ratios are similar (2.3–2.7). We hypothesize that in comparison with **D5B2** and **D5B2-1/2NaOH**, which were polymerized with 0 and 0.5 eq. of NaOH, respectively, the protonated DMAPM groups (ammoniums) within **D5B2-1/1HCl** and **D5B2-1/2HCl** are relatively distant from the hydrophobic pNIPAm moieties and backbones in the collapsed GPs. This is because the protonated amine groups are relatively polar and hydrophilic. In other words, the amine groups of the GPs prepared with HCl are located in a “pseudo-swollen” microenvironment analogous to the swollen state even at the collapsed state. After the volume phase transition from the collapsed to the swollen state, swelling has less effect on the amines within the “pseudo-swollen” GPs. As a result, the Δp*K*_a_ values of the GPs prepared with HCl are less than those of the GPs prepared without HCl.

The reversible CO_2_ capture stoichiometries of the GP films are presented as red plots in Fig. 1a. **D5B2-1/1HCl** and **D5B2-1/2HCl** show very low reversible CO_2_ capture stoichiometries (0.18 and 0.27 mol CO_2_ per mol amine, respectively), while the values for **D5B2** and **D5B2-1/2NaOH** are both ∼0.97 mol CO_2_ per mol amine.

**Scheme 2:**
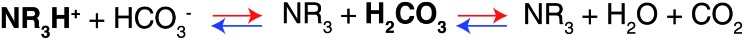
Equilibrium of the tertiary amine-CO_2_ reaction.

The difference in the stoichiometry can be interpreted by the acid–base theory as follows. It has been reported that the tertiary amines in GPs and CO_2_ form ammonium ions (RN_3_H^+^) and bicarbonate ions (HCO_3_^–^) *via* base-catalyzed reactions.^44^,^45^ In aqueous media containing RN_3_H^+^ and HCO_3_^–^, there is always an equilibrium between their corresponding conjugated base–acid, as described in Scheme 2.

When the p*K*_a_ of NR_3_H^+^ is above the p*K*_a1_ of H_2_CO_3_, which indicates that NR_3_H^+^ holds a proton more tightly than H_2_CO_3_ does, the proton will move from the stronger acid, H_2_CO_3_, to the stronger base, NR_3_. As a result, more R_3_N will be consumed and more CO_2_ will be captured, as presented by the blue arrows in Scheme 2. In contrast, when the p*K*_a_ of NR_3_H^+^ is below the p*K*_a1_ of H_2_CO_3_, more CO_2_ will be released, as indicated by the red arrows in Scheme 2.^46^

The p*K*_a1_ value of carbonic acid (H_2_CO_3_) is in the range of 6.35 ± 0.05 at both 30 °C and 75 °C, as determined by Harned and Davis,^47^ however, the p*K*_a_ values of the amine-containing thermal responsive GPs dramatically depend on the temperature, as presented in Fig. 1. The high p*K*_a_ values of **D5B2-1/1HCl** and **D5B2-1/2HCl** at 75 °C (7.4 and 7.0, respectively) are higher than the p*K*_a1_ of H_2_CO_3_ (6.35) and inhibit the release of CO_2_. As a result, the reversible CO_2_ capture stoichiometries of **D5B2-1/1HCl** and **D5B2-1/2HCl** are low (0.18 and 0.27 mol CO_2_ per mol amine, respectively), although the p*K*_a_ values at 30 °C (8.9 and 8.6, respectively) are high enough to capture CO_2_ efficiently. For **D5B2** and **D5B2-1/2NaOH**, the high reversible CO_2_ capture stoichiometries (0.97 mol CO_2_ per mol amine for both) are achieved due to the low p*K*_a_ values at 75 °C (5.3 and 4.9, respectively), which are below the p*K*_a1_ of H_2_CO_3_, and meanwhile, the high p*K*_a_ values at 30 °C (7.8 and 7.2, respectively), which are above the p*K*_a1_ of H_2_CO_3_.

### Effect of Δp*K*_a_ values of GPs on the reversible CO_2_ capture stoichiometry of GP films

Despite the low p*K*_a_ values of **D5B2** and **D5B2-1/2NaOH** at 75 °C (Fig. 1a), their larger Δp*K*_a_ values between 30 °C and 75 °C (2.5 and 2.3, respectively) than those of **D5B2-1/1HCl** and **D5B2-1/2HCl** (1.5 and 1.6, respectively) may also be responsible for their high reversible CO_2_ capture stoichiometries.

In order to distinguish the effects of the p*K*_a_ value and the Δp*K*_a_ on the reversible CO_2_ capture stoichiometry of the GP films, a GP with a smaller Δp*K*_a_ than **D5B2** was prepared by increasing the Bis cross-linker to 10 mol% into the GP that contains 5 mol% DMAPM (**D5B10**), since it has been reported that the crosslink degree influences the Δp*K*_a_ of the GPs.^24^

**Fig. 2:**
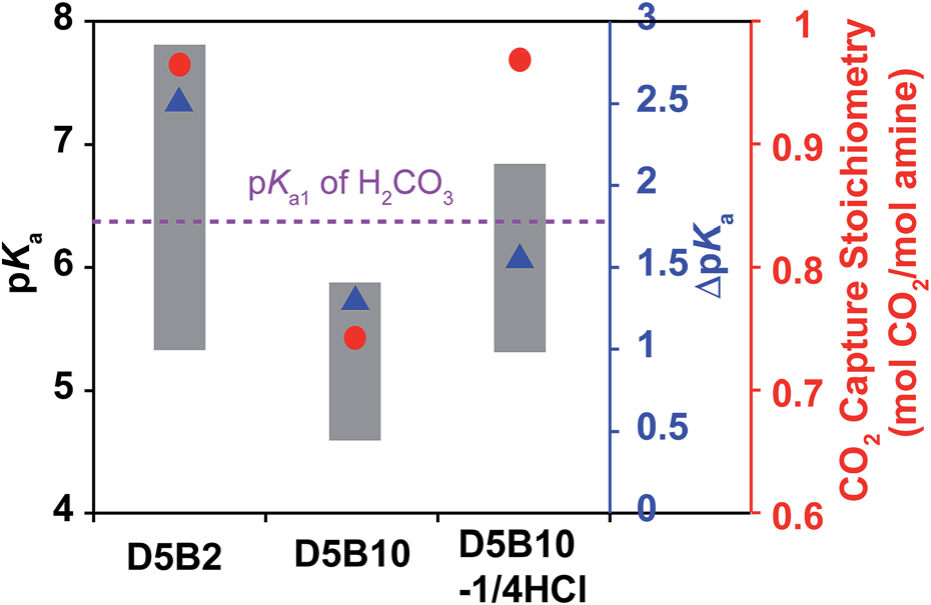
p*K*_a_ values (left *Y*-axis) at 75 °C (bottom of bar) and 30 °C (top of bar), and Δp*K*_a_ values (blue right *Y*-axis, blue triangle) between 75 °C and 30 °C of **D5B2**, **D5B10**, and **D5B10-1/4HCl**, reversible CO_2_ capture stoichiometries (red right *Y*-axis, red dot) of GP films in the temperature range 30–75 °C.

The p*K*_a_ values of **D5B2** and **D5B10** at 30 °C and 75 °C are plotted as the top and bottom of the gray bars in Fig. 2. The Δp*K*_a_ of **D5B10** (1.4) is apparently less than that of **D5B2** (2.5). However the p*K*_a_ value of **D5B10** (5.9) at 30 °C is below the p*K*_a1_ of H_2_CO_3_. Thus the possible reason for the lower reversible CO_2_ capture stoichiometry of **D5B10** than **D5B2** (0.74 and 0.97 mol CO_2_ per mol amine, respectively), the smaller Δp*K*_a_ of **D5B10** than **D5B2** and/or the low p*K*_a_ value of **D5B10** at 30 °C which is below the p*K*_a1_ of H_2_CO_3_, still cannot be distinguished.

To identify the main factor that governs the reversible CO_2_ capture stoichiometry, we designed another GP with a greater p*K*_a_ value than **D5B10** and a lower Δp*K*_a_ than **D5B2**, using the “microenvironment-imprinting” strategy described above. The GPs were prepared by polymerizing **D5B10** in the presence of an appropriate amount of HCl (**D5B10-1/4HCl**).

Fig. 2 shows the p*K*_a_ values and Δp*K*_a_ of **D5B10-1/4HCl**. The p*K*_a_ values at 30 °C and 75 °C of **D5B10-1/4HCl** (6.8 and 5.3, respectively) are higher than those of **D5B10** (5.9 and 4.5, respectively), but the Δp*K*_a_ of **D5B10-1/4HCl** (1.5) is comparable to that of **D5B10** (1.4). **D5B10-1/4HCl** shows a much higher CO_2_ capture stoichiometry (0.97 mol CO_2_ per mol amine) than **D5B10** (0.74 mol CO_2_ per mol amine), despite the similar Δp*K*_a_ values.

On the other hand, as displayed in Fig. 2, the p*K*_a_ value of **D5B10-1/4HCl** at 75 °C (5.3) is similar to that of **D5B2** (5.3), while the Δp*K*_a_ of **D5B10-1/4HCl** (1.5) is much less than that of **D5B2** (2.5). However, both GPs exhibit comparably high reversible CO_2_ capture stoichiometries (0.97 mol CO_2_ per mol amine), even though the Δp*K*_a_ values are much different.

We conclude from these results that the Δp*K*_a_ has a minor influence on the reversible CO_2_ capture stoichiometry, and a high reversible CO_2_ capture stoichiometry is possible for GPs that exhibit reduced Δp*K*_a_ values as long as the p*K*_a_ values lie within the appropriate range. The p*K*_a_ value of the GPs must be tuned above the p*K*_a1_ of H_2_CO_3_ (6.35) at the CO_2_ capture temperature (30 °C) and below the p*K*_a1_ of H_2_CO_3_ (6.35) at the CO_2_ release temperature (75 °C) in order to achieve a high reversible CO_2_ capture stoichiometry.

### Effect of VPTT of GPs on the reversible CO_2_ capture stoichiometry of GP films

As shown in Fig. 1b, the thermal responsive volume phase transition of the GPs leads to a sharp p*K*_a_ transition. In addition, the p*K*_a_ value of the GPs has been revealed essential for the reversible CO_2_ capture stoichiometry. Therefore, it is interesting to observe the effect of the VPTT of GPs on the reversible CO_2_ capture stoichiometry of the GP films.

**Fig. 3:**
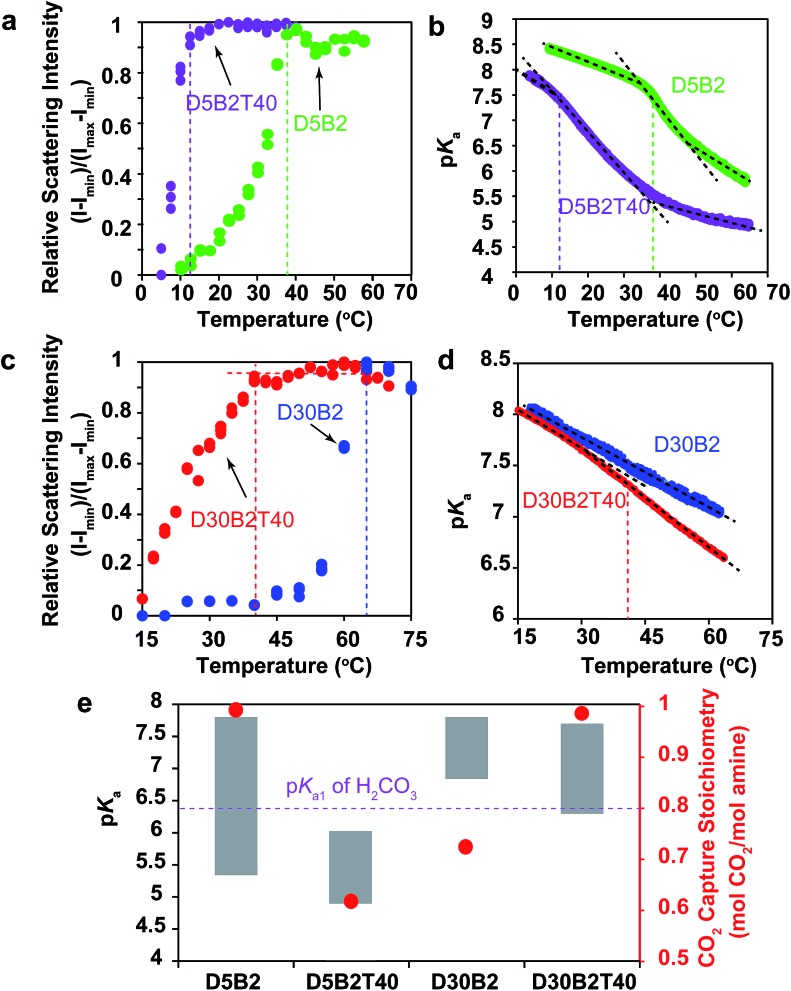
(a) Relative scattering intensity as a function of temperature for **D5B2** (green) and **D5B2T40** (purple). (b) p*K*_a_ shift of **D5B2** (green) and **D5B2T40** (purple) during the heating process. (c) Relative scattering intensity as a function of temperature for **D30B2** (blue) and **D30B2T40** (red). (d) p*K*_a_ shift of **D30B2** (blue) and **D30B2T40** (red) during heating process. (e) p*K*_a_ values (left *Y*-axis) at 75 °C (bottom of bar) and 30 °C (top of bar) of the GPs, and reversible CO_2_ capture stoichiometries (right *Y*-axis, red dot) of the GP films in the temperature range 30–75 °C.

A GP with a lower VPTT than **D5B2** was synthesized by polymerizing 40 mol% of TBAm, which is more hydrophobic than NIPAm, together with 5 mol% DMAPM and 2 mol% Bis (**D5B2T40**). The relative scattering intensity of the GP solutions is shown in Fig. 3a. Compared with **D5B2** (VPTT = 38 °C), the VPTT of **D5B2T40** is only 12 °C. The higher intra-particle hydrophobic interaction of the GPs containing 40 mol% TBAm causes the entropy-driven collapse of **D5B2T40** to occur at a lower temperature.^48^,^49^

Fig. 3b shows the temperature dependent p*K*_a_ values of **D5B2** and **D5B2T40**. Similar to **D5B2**, **D5B2T40** shows a sharp p*K*_a_ transition at a temperature around its VPTT (12 °C) and a steep p*K*_a_ decrease continues over a wide temperature range above the VPTT. As a result, the p*K*_a_ values of **D5B2T40** at 30 °C and 75 °C (6.0 and 4.8, respectively) are both low. The lower reversible CO_2_ capture stoichiometry of **D5B2T40** (0.60 mol CO_2_ per mol amine) than that of **D5B2** (0.97 mol CO_2_ per mol amine), can be explained by the low p*K*_a_ value of **D5B2T40** at 30 °C (6.0), which is below the p*K*_a1_ of H_2_CO_3_.

Besides **D5B2** and **D5B2T40**, another pair of GPs with different VPTTs were prepared by increasing the feed ratio of DMAPM to 30 mol% (**D30B2** and **D30B2T40**).^48^,^49^ Fig. 3c shows that the VPTT of **D30B2** is above 60 °C. To lower the VPTT, 40 mol% of TBAm was incorporated into the 30 mol% DMAPM-containing GP (**D30B2T40**). In comparison, the VPTT of **D30B2T40** is only 40 °C (Fig. 3c).

In Fig. 3d, the p*K*_a_ value of **D30B2** is linearly dependent on temperature, and no obvious sharp transition is observed in the temperature range due to the high VPTT. However, the p*K*_a_ value of **D30B2T40** exhibits a transition from the temperature around its VPTT (40 °C), and a steeper p*K*_a_ decrease than that of **D30B2** is observed at the temperatures above the VPTT. As a result, **D30B2T40** has lower p*K*_a_ values at higher temperatures (>40 °C) and a larger Δp*K*_a_ between 30 °C and 75 °C than **D30B2** (Fig. 3e).

The degree of p*K*_a_ transition of **D30B2T40** (Fig. 3d) induced by the phase transition is less significant than **D5B2** and **D5B2T40** (Fig. 3b). This may be a result of the increased intra-GP charge repulsion within **D30B2T40** caused by the smaller amine–amine distance.

The p*K*_a_ values at 30 °C and 75 °C of **D30B2** (7.8 and 6.8, respectively) and **D30B2T40** (7.7 and 6.3, respectively) are shown in Fig. 3e. The reversible CO_2_ capture stoichiometry (0.72 mol CO_2_ per mol amine) of **D30B2** is quite low; however, that of **D30B2T40** is much higher at 0.97 mol CO_2_ per mol amine. The improved stoichiometric efficiency can be explained by the relatively low p*K*_a_ value of **D30B2T40** at 75 °C (6.3), which is below the pK_a1_ of H_2_CO_3_. The VPTT of **D30B2T40**, which is close to 30 °C, induces a sharp p*K*_a_ transition and a steep p*K*_a_ decrease over a wide temperature range above the VPTT, lowers the p*K*_a_ value at 75 °C, and consequently improves the reversible CO_2_ capture stoichiometry.

In summary, the results in Fig. 3 lead to a comparable conclusion to those of Fig. 1 and 2: the p*K*_a_ values of the GPs at 30 °C and 75 °C govern the reversible CO_2_ capture stoichiometry. However, the VPTT of the GPs also plays a crucial role. The GPs with a much lower VPTT than the CO_2_ capture temperature (30 °C) show low reversible CO_2_ capture stoichiometries due to the low p*K*_a_ value. Meanwhile, to achieve a high reversible CO_2_ capture stoichiometry, the VPTT should be above and as close to 30 °C as possible to generate a large p*K*_a_ transition over a wide temperature range above the VPTT.

However, a VPTT above and close to 30 °C is not enough for a high CO_2_ capture stoichiometry if the p*K*_a_ values at 30 °C and 75 °C lie at an improper level. For example, in Fig. 1, though the VPTTs of **D5B2-1/1HCl** and **D5B2-1/2HCl** are both 38 °C, the high p*K*_a_ values at 75 °C (7.4 and 7.0, respectively) considerably restrict efficient CO_2_ release (0.18 and 0.27 mol CO_2_ per mol amine, respectively).

### Effect of GP size on the reversible CO_2_ capture stoichiometry of GP films

We have reported that the GP films exhibited larger CO_2_ capture capacities than conventional bulk hydrogel films due to the small dimensions of GPs, which lead to a fast response and fast ion diffusion.^29^ Herein, the effect of GP size on the reversible CO_2_ capture stoichiometry is discussed.

Larger GPs can be prepared by increasing the concentration of monomer or by decreasing the concentration of surfactant.^50^ In this study, GPs with the same composition (5 mol% DMAPM and 2 mol% Bis) but different size were designed. A GP with a larger hydrodynamic diameter (**D5B2-L**) than **D5B2** was prepared by decreasing the concentration of surfactant, while a smaller GP (**D5B2-S**) was synthesized using a monomer solution with a lower total concentration.

**Fig. 4:**
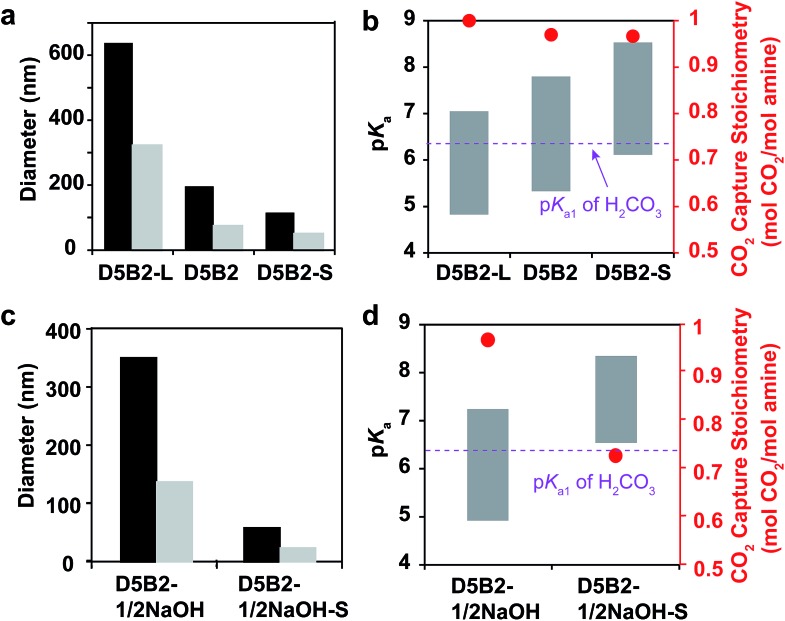
(a) Diameters at 30 °C (black bar) and 75 °C (gray bar) of **D5B2-L**, **D5B2**, and **D5B2-S**. (b) p*K*_a_ values (left *Y*-axis) at 75 °C (bottom of bar) and 30 °C (top of bar) of the GPs, and the reversible CO_2_ capture stoichiometries (right *Y*-axis, red dot) of the GP films in the temperature range 30–75 °C. (c) Diameters at 30 °C (black bar) and 75 °C (gray bar) of **D5B2-1/2NaOH** and **D5B2-1/2NaOH-S**. (d) p*K*_a_ values (left *Y*-axis) at 75 °C (bottom of bar) and 30 °C (top of bar) of the GPs, and the reversible CO_2_ capture stoichiometries (right *Y*-axis, red dot) of the GP films in the temperature range 30–75 °C.

The diameters of the GPs at 30 °C and 75 °C are shown in Fig. 4a. As expected, **D5B2-L** (638 nm at 30 °C and 322 nm at 75 °C) has a larger diameter than **D5B2** (196 nm at 30 °C and 74 nm at 75 °C), and **D5B2-S** has the smallest diameter (115 nm at 30 °C and 49 nm at 75 °C). The p*K*_a_ values of the GPs at 30 °C and 75 °C are presented as the top and bottom, respectively, of the bars in Fig. 4b. It can be seen clearly that the p*K*_a_ values of the GPs at both temperatures increase with the decreasing diameter.

The amine groups located at the exterior of the GPs are expected to show higher p*K*_a_ values than those of the interior groups because of the high dielectric constant of water, as well as the reduced steric hindrance. In the meantime, the amine groups of the exterior of the smaller GP account for a much larger percentage than those of the larger GPs. Therefore, smaller GPs show higher p*K*_a_ values than the larger GPs.

Fig. 4b also shows that the three GPs exhibit similar, high reversible CO_2_ capture stoichiometries (0.96–1.0 mol CO_2_ per mol amine), despite the difference in GP diameters and p*K*_a_ values.

Another pair of GPs showing different p*K*_a_ ranges from those of the GPs in Fig. 4a and b was polymerized using the “microenvironment-imprinting” strategy by adding 0.5 eq. of NaOH to the monomer solution (**D5B2-1/2NaOH** and **D5B2-1/2NaOH-S**). **D5B2-1/2NaOH-S**, having a smaller diameter than **D5B2-1/2NaOH**, was prepared by lowering the total monomer concentration.

Fig. 4c and d show the diameters and p*K*_a_ values, respectively, of the GPs at 30 °C and 75 °C. In good agreement with the results in Fig. 4a, the diameters of **D5B2-1/2NaOH-S** (83 nm at 30 °C and 57 nm at 75 °C) are much smaller than the diameters of **D5B2-1/2NaOH** (715 nm at 30 °C and 349 nm at 75 °C). The p*K*_a_ values of the smaller GP are also higher than those of the larger GP. However, the reversible CO_2_ capture stoichiometry of **D5B2-1/2NaOH-S** (0.74 mol CO_2_ per mol amine) is lower than that of **D5B2-1/2NaOH** (0.98 mol CO_2_ per mol amine), as shown in Fig. 4d.

The different results from Fig. 4b and d could also be ascribed to the different p*K*_a_ values of the GPs. The high p*K*_a_ values of **D5B2-L**, **D5B2**, **D5B2-S**, and **D5B2-1/2NaOH** at 30 °C (7.0, 7.8, 8.5, and 7.2, respectively), which are above the p*K*_a1_ of H_2_CO_3_ (6.35), and the low p*K*_a_ values at 75 °C (4.8, 5.3, 6.1, and 4.9, respectively), which are below the p*K*_a1_ of H_2_CO_3_, result in high reversible CO_2_ capture stoichiometries. However, the p*K*_a_ values of **D5B2-1/2NaOH-S** at 30 °C and 75 °C are 8.4 and 6.6, respectively. Its high p*K*_a_ value at 75 °C (6.6), which is above the p*K*_a1_ of H_2_CO_3_, restricts the efficient release of CO_2_, resulting in a low reversible CO_2_ capture stoichiometry (0.74 mol CO_2_ per mol amine).

In general, the results of Fig. 4 lead to the conclusion that the reversible CO_2_ capture stoichiometry of GPs can be improved by regulating their p*K*_a_ values at both 30 °C and 75 °C through varying their diameter. The smaller diameter leads to a higher p*K*_a_ value.

### Effect of swelling ratio of GP on the reversible CO_2_ capture stoichiometry of GP films

The swelling ratio of thermal responsive GPs plays an important role in their function such as the reversible target binding.^26^,^51^,^52^ The swelling ratio of GPs is typically controlled by the crosslink degree.^53^ Herein, we prepared a series of GPs containing 5 mol% of DMAPM with 0, 2, 5, and 10 mol% Bis crosslinker (**D5B0**, **D5B2**, **D5B5**, and **D5B10**, respectively) to investigate the effect of the swelling ratio on the reversible CO_2_ capture stoichiometry of GPs.

**Fig. 5:**
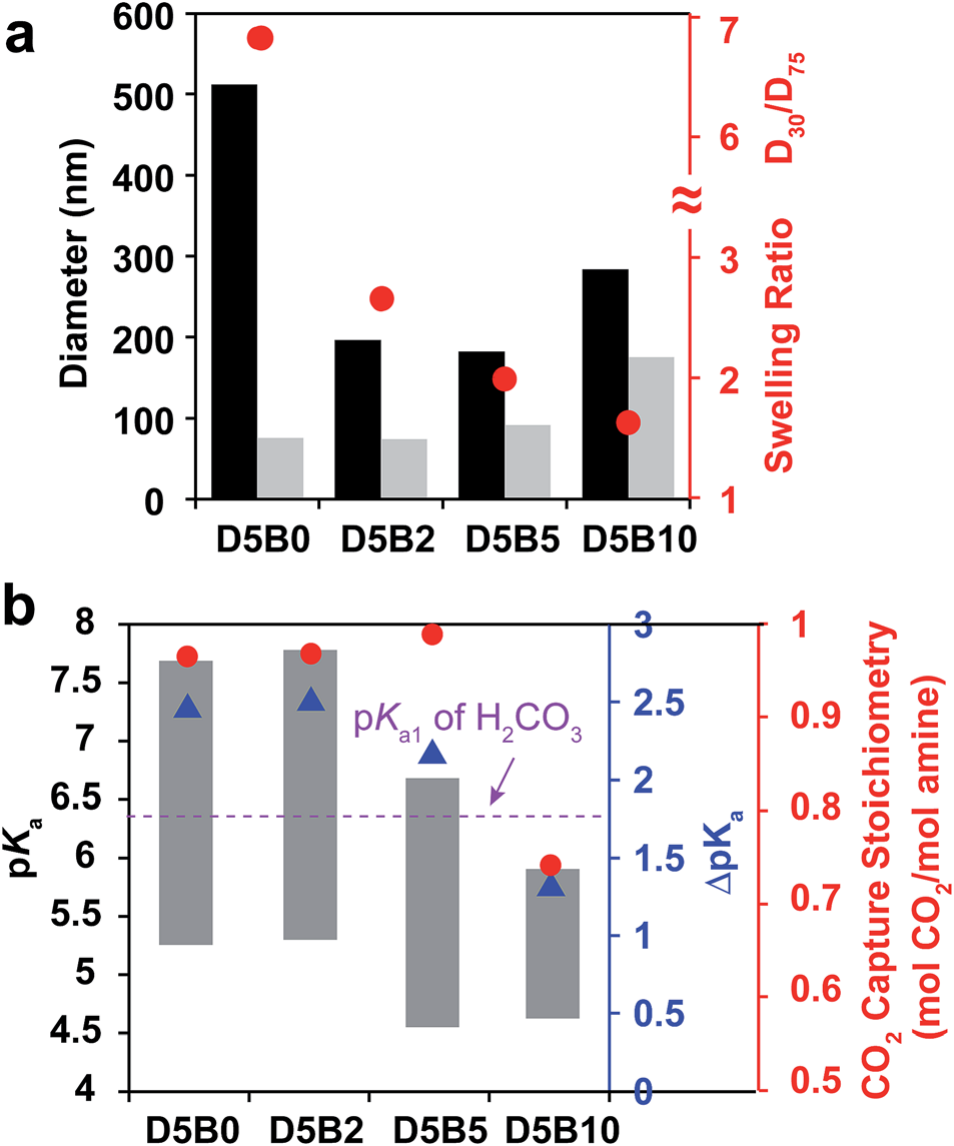
(a) Diameters (left *Y*-axis) at 30 °C (black bar) and 75 °C (gray bar) and swelling ratios (right *Y*-axis, red dot) between 30 °C and 75 °C of **D5B0**, **D5B2**, **D5B5**, and **D5B10**. (b) p*K*_a_ values (left *Y*-axis) at 75 °C (bottom of bar) and 30 °C (top of bar), and Δp*K*_a_ values (blue right *Y*-axis, blue triangle) between 75 °C and 30 °C of **D5B0**, **D5B2**, **D5B5**, and **D5B10**, reversible CO_2_ capture stoichiometries (red right *Y*-axis, red dot) of the GP films in the temperature range 30–75 °C.

The diameters at 30 °C and 75 °C, and the swelling ratios of the GPs are plotted in Fig. 5a. It is noteworthy that GP can be obtained without Bis crosslinker (**D5B0**), possibly due to the slight self-crosslinking.^54^ As expected, the swelling ratios of **D5B2** (2.7), **D5B5** (2.0), and **D5B10** (1.6) decrease with the increasing crosslink degree. The diameters of the GPs at 75 °C increase slightly with the increasing amount of Bis crosslinker, indicating that Bis influences the nucleation process during polymerization, as observed by Pelton's group.^50^

Fig. 5b shows the p*K*_a_ values of the GPs at 30 °C and 75 °C, and the Δp*K*_a_ values. The Δp*K*_a_ values of **D5B2** (2.5), **D5B5** (2.1), and **D5B10** (1.3) between 30 °C and 75 °C show a positive correlation with the swelling ratios of the GPs (2.7, 2.0, and 1.6, respectively). This suggests that from 30 °C to 75 °C the microenvironmental changes, such as the change of dielectric constant and polymer density, around the less cross-linked amines are greater than those of the highly cross-linked amines in the GPs. At 30 °C, the p*K*_a_ values of the swollen GPs, **D5B2** (7.8), **D5B5** (6.7), and **D5B10** (5.9), decrease with the reduction in the swelling ratio, indicating the GP with a smaller swelling ratio is in a relatively collapsed state at 30 °C compared to those with higher swelling ratios, because swelling of the GP is inhibited by the high crosslink degree. The p*K*_a_ value of **D5B2** at 30 °C (7.8) is similar to that of **D5B0** (7.7), although **D5B0** exhibits a much larger swelling ratio, indicating that **D5B2** is also fully swollen at 30 °C.

In Fig. 5b, **D5B0**, **D5B2**, and **D5B5** exhibit similar reversible CO_2_ capture stoichiometries (0.97–1.0 mol CO_2_ per mol amine), while that of **D5B10** (0.74 mol CO_2_ per mol amine) is much lower. The difference in the reversible CO_2_ capture stoichiometries can also be explained by their p*K*_a_ values. The higher p*K*_a_ values of **D5B0**, **D5B2**, and **D5B5** at 30 °C (7.7, 7.8, and 6.7, respectively), which are above the p*K*_a1_ of H_2_CO_3_, and the lower p*K*_a_ values at 75 °C (5.2, 5.3, and 4.4, respectively), which are below the p*K*_a1_ of H_2_CO_3_, lead to high reversible CO_2_ capture stoichiometries. However, the p*K*_a_ value of **D5B10** at 30 °C (5.9) is too low and the basicity is too weak to capture CO_2_ efficiently.

Conclusively, the swelling ratio (*D*_30_/*D*_75_), which can be controlled by crosslink degree, is an important factor to tune the Δp*K*_a_ and consequently the p*K*_a_ values of GPs to improve the reversible CO_2_ capture stoichiometry.

### Design rationale of GP film with large reversible CO_2_ capture stoichiometry

The above discussion has revealed the design rationale of GPs that show large reversible CO_2_ capture stoichiometry within a narrow temperature range of 30 °C to 75 °C. The p*K*_a_ values of the GPs at 30 °C and 75 °C are the principal factors that govern the reversible CO_2_ capture stoichiometry. Higher p*K*_a_ values at 30 °C, above the p*K*_a1_ of H_2_CO_3_ (6.35), and lower p*K*_a_ values at 75 °C, below the p*K*_a1_ of H_2_CO_3_, enable high reversible CO_2_ capture stoichiometries of GP films. This is because the GPs are capable of capturing CO_2_ efficiently at 30 °C due to the stronger basicity of the amine than HCO_3_^–^, and then releasing CO_2_ sufficiently at 75 °C because of the weaker basicity. High reversible CO_2_ capture stoichiometry can be achieved for GPs that exhibit a smaller Δp*K*_a_, as long as the p*K*_a_ values lie within the appropriate range.

**Scheme 3:**
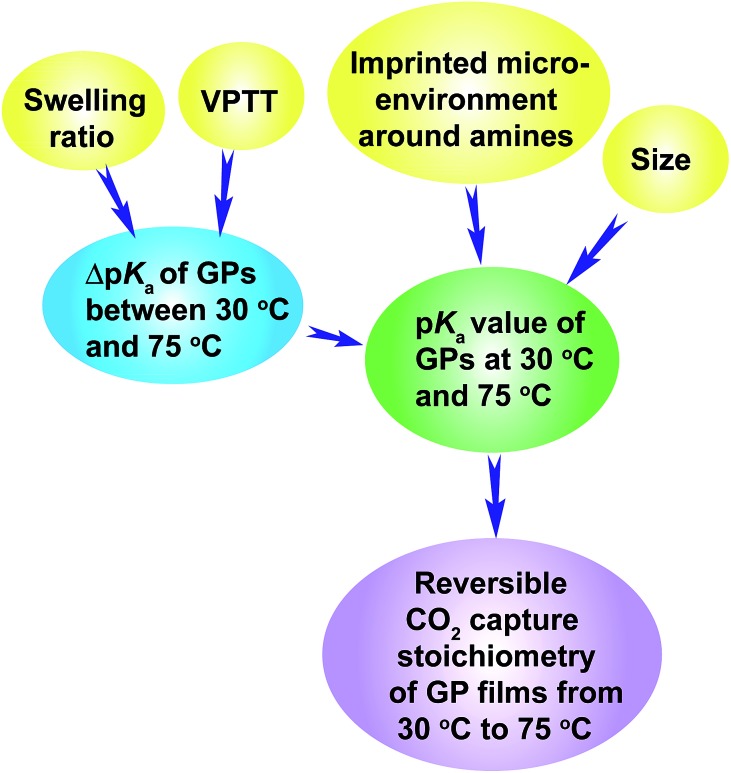
Illustration of the design rationale for GPs that show large reversible CO_2_ capture stoichiometry.

The p*K*_a_ value of the GPs can be readily adjusted to the desired level by varying the VPTT of the GPs above and close to the CO_2_ capture temperature (30 °C in this study), because the volume phase transition of GPs always brings out a large p*K*_a_ transition throughout a wide temperature range above the VPTT. The p*K*_a_ value of the GPs can also be tuned by controlling the size of the GPs since smaller GPs show higher p*K*_a_ values. Another method is to regulate the GP swelling ratio, which influences Δp*K*_a_ and consequently the p*K*_a_ value of GPs. Finally, the imprinted microenvironment around the amine groups in the GPs can also influence the p*K*_a_ values of the GPs because the GPs synthesized in the presence of a large amount of protons exhibit higher p*K*_a_ values. The inter-relationship between these factors is illustrated by Scheme 3.

### Optimization of GPs to maximize reversible CO_2_ capture capacity

**Fig. 6:**
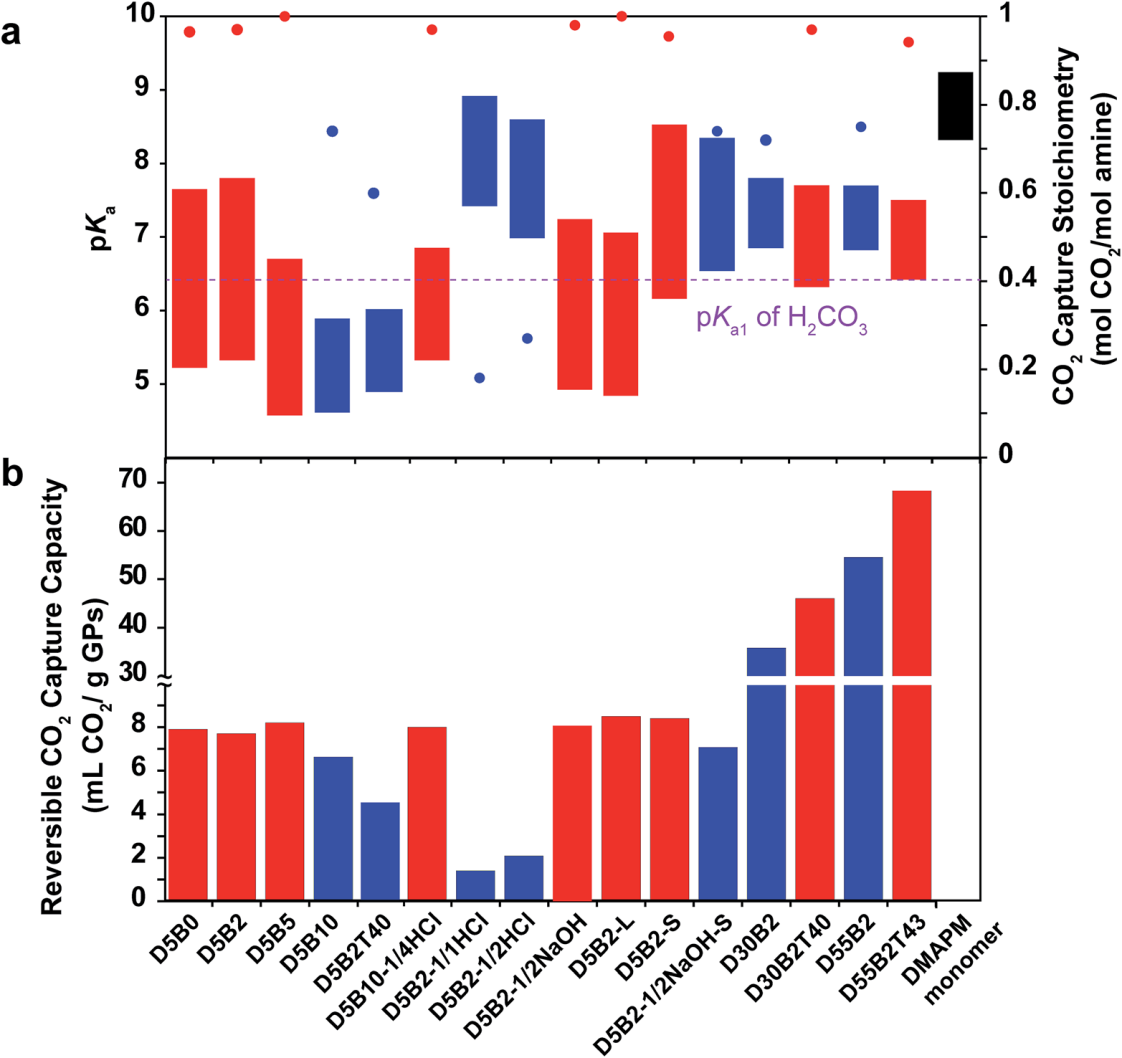
(a) p*K*_a_ values (left *Y* axis) at 75 °C (bottom of bar) and 30 °C (top of bar) of the GPs studied in this paper, and the reversible CO_2_ capture stoichiometries (right *Y* axis, red dot) of the GP films in the temperature range 30–75 °C. (b) Reversible CO_2_ capture capacities of the GP films in the temperature range 30–75 °C. Red represents GPs with high reversible CO_2_ capture stoichiometries (>0.9 mol CO_2_ per mol amine) and blue represents those with low stoichiometries (<0.75 mol CO_2_ per mol amine).

In order to improve the CO_2_ capture capacity by GP films, the ratio of functional amine monomer, DMAPM, was maximized to 55 mol% (GPs with a higher DMAPM concentration precipitated during polymerization process). However, **D55B2** exhibited a low CO_2_ capture stoichiometry (0.73 mol CO_2_ per mol amine) due to its high p*K*_a_ value at 75 °C (6.8) (Fig. 6a). According to the design rationale described above, highly efficient CO_2_ capture cycles could be achieved if the p*K*_a_ value of the 55 mol% DMAPM-containing GP at 75 °C is below the p*K*_a1_ of H_2_CO_3_ (6.35).

Since the VPTT of **D55B2** is greater than 75 °C, one approach to reducing the p*K*_a_ value of the 55 mol% DMAPM-containing GP at 75 °C is to lower its VPTT. Thus, 43 mol% TBAm was incorporated into the 55 mol% DMAPM-containing GP (**D55B2T43**).

In accordance with the design rationale, the p*K*_a_ value of **D55B2T43** at 75 °C decreases to about 6.35 (Fig. 6a) due to the reduced VPTT *via* the incorporation of TBAm. Consequently, a high reversible CO_2_ capture stoichiometry (0.93 mol CO_2_ per mol amine) is obtained. As a result of the high amine content and the high stoichiometric efficiency, as shown in Fig. 6b, **D55B2T43** shows the largest reversible CO_2_ capture capacity (68 mL CO_2_ per g dry GPs, 3.0 mmol CO_2_ per g dry GPs) in this study.

Though the GP **D55B2T43** showed high CO_2_ capture capacity (68 mL CO_2_ per g dry GPs, 3.0 mmol CO_2_ per g dry GPs), taking into account of water and support that are necessary for the GP films, the CO_2_ capture capacity of the GP film is lower than the best adsorbents.^55^ However the GP films can be used directly to capture CO_2_ from desulfurized post-combustion exhausted gas, without pre-treatment of the exhausted gas to remove water vapor inside, nor to decrease the temperature of the water vapor. Furthermore, the low regeneration temperature of the GP films (75 °C) enables the utilization of abundant and low cost waste heat (<100 °C) of factories as an energy source. Consequently the energy consumption of the GP films might be lowered.

Fig. 6a summarizes the p*K*_a_ values and the reversible CO_2_ capture stoichiometries of the GPs studied in this paper. Overall, it can be concluded that the GPs with p*K*_a_ values at 30 °C above the p*K*_a1_ of H_2_CO_3_, and p*K*_a_ values at 75 °C below the p*K*_a1_ of H_2_CO_3_ (red) generally show larger reversible CO_2_ capture stoichiometries than other GPs (blue).

### Reversibility of the optimized GPs as CO_2_ absorbent in wet environment

**Fig. 7:**
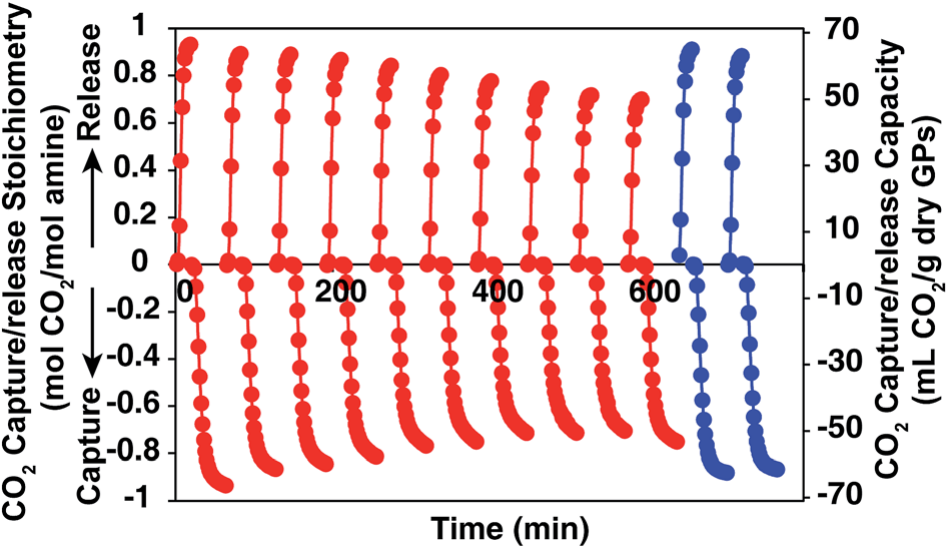
CO_2_ capture/release stoichiometry (right *Y* axis) and CO_2_ capture/release capacity (left *Y* axis) of GP **D55B2T43** film with 4 mL water per g GPs. Red: first 10 cycles of CO_2_ release/capture by the GP film. After the first 10 cycles, 4 mL water per g GPs was supplied to the dried part of the GP film. Blue: the CO_2_ release-capture by the GP film after supplying 4 mL water per g GPs to the dried part of the GP film.

In order to show the cycle stability of the GP film, the reversible CO_2_ capture-release by **D55B2T43** film was carried out for 10 cycles. The result is shown in Fig. 7 red plots. The reversible CO_2_ capture capacity decreased gradually from 0.93 mol CO_2_ per mol amine of the 1^st^ cycle to 0.71 mol CO_2_ per mol amine of the 10^th^ cycle. The reason is that the GP film partly dried out, as can be seen in Fig. 2S in ESI.† However, by supplying 4 mL water per g GPs to the dried part of the GP film after 10 CO_2_ release/capture cycles, the reversible CO_2_ capture capacity can be completely recovered (Fig. 7 blue plots).

We believe the problem of cycle stability can be solved by tuning mass balance of water carefully by engineering the CO_2_ capture processes, such as reactor shape and operation condition optimization.

## Conclusions

Inspired by the efficient CO_2_ transport mechanism of hemoglobin, known as the Bohr effect, here we revealed the design rationale of amine-functionalized thermo-responsive gel particle (GP) films that reversibly capture and release large amounts of CO_2_ efficiently from a model of the exhaust gas of fire power plants over a narrow temperature range (30–75 °C). Appropriate p*K*_a_ values of the GPs at the CO_2_ capture and release temperatures (30 °C and 75 °C, respectively) are essential for the reversible CO_2_ capture stoichiometry of the GPs films. At 30 °C, a high p*K*_a_ value, above the p*K*_a1_ of H_2_CO_3_, is required for efficient CO_2_ capture. Simultaneously, a low p*K*_a_ value at 75 °C, below the p*K*_a1_ of H_2_CO_3_, is required for the efficient release of CO_2_.

The p*K*_a_ value of the GPs can be readily adjusted to the desired level *via* four methods. (1) Controlling the VPTT of the GPs above and close to 30 °C – the phase transition induces a large p*K*_a_ transition over a wide temperature range above the VPTT. (2) Controlling the size of the GPs – smaller GPs show higher p*K*_a_ values. (3) Controlling the swelling ratio, which influences Δp*K*_a_, and consequently the p*K*_a_ value of the GPs. (4) Controlling the imprinted microenvironment of the GPs – the GPs synthesized in the presence of a large amount of protons exhibit higher p*K*_a_ values.

We successfully designed and acquired the GP, **D55B2T43**, which exhibited a large reversible CO_2_ capture capacity (68 mL CO_2_ per g dry GPs, 3.0 mmol CO_2_ per g dry GPs) as well as a high reversible CO_2_ capture stoichiometry (0.93 mol CO_2_ per mol amine), by optimizing the p*K*_a_ value of GPs containing a maximum amount of amine-monomer. We believe GPs with an even larger CO_2_ capture capacity could be designed by incorporating co-monomers that are more hydrophobic than TBAm into the tertiary amine-containing GPs, and/or by using more hydrophobic tertiary amine monomers such as *N*-[3-(diethylamino)propyl] methacrylamide. The advantages of amine functionalized GP films, such as the large capture capacity, the low regeneration temperature (75 °C), and the unique ability to work in wet environments, would enable the use of GP films as energy efficient CO_2_ absorbents for the exhaust gas of fire power plants. We anticipate that GP films that can reversibly capture other acidic and basic gases with a large capacity can also be achieved by the same strategy inspired by the Bohr effect of hemoglobin.

## Supplementary Material

Supplementary information
