# Supplementary material for: Design rationale of thermally responsive microgel particle films that reversibly absorb large amounts of CO2: fine tuning the pKa of ammonium ions in the particles
Source: Chem Sci. 2015 Jul 28;6(11):6112–23. doi: 10.1039/c5sc01978h (PMC6054111; doi:10.1039/c5sc01978h)
Supplement: Supplementary file 1 [file SC-006-C5SC01978H-s001.pdf]

Electronic Supplementary Material (ESI) for Chemical Science.
This journal is © The Royal Society of Chemistry 2015

Electronic Supplementary Information (ESI) for Chemical Science.

Supporting Information

Design Rationale of Thermally Responsive Microgel Particle Films That Reversibly Absorb Large Amounts of CO_2: Fine Tuning the pK_a of Ammonium Ions in the Particles

Mengchen Yue, Yu Hoshino*, Yoshiko Miura

Department of Chemical Engineering, Kyushu University, 744 Motooka, Nishi-ku, Fukuoka 819-0395, Japan

To whom correspondence may be addressed: yhoshino@chem-eng.kyushu-u.ac.jp

1. Chemicals and materials

NIPAm (Wako Pure Chemical Industries, Ltd.) was purified by recrystallization from *n*-hexane and dried *in vacuo* at room temperature. *N*-[3-(dimethylamino)propyl]methacrylamide (DMAPM, Tokyo Chemical Industry Co., Ltd.) was purified using an Al_2O_3 column (Merck KGaA) prior to use. 2,2-Azobis(2-methylpropionamidine) dihydrochloride (AAPD, Wako Pure Chemical Industries, Ltd.), *N,N*-methylenebis(acrylamide) (Bis, Tokyo Chemical Industry Co., Ltd.), cetyltrimethylammonium bromide (CTAB, Wako Pure Chemical Industries, Ltd.) were used as received. The dialysis membrane (MWCO 12,000–14,000) was purchased from Spectrum Laboratories, Inc. The anion-exchange resin (A2004-OH, Muromachi Technos Co., Ltd.) was first activated by 1 M NaOH, and then washed carefully with deionized water. The water was purified using a Direct-Q Ultrapure Water System (Merck, Ltd.).

2. Preparation of GPs

According to the different polymerization conditions of each GP described in Table 1, the monomers were first dissolved in water to achieve the specified total concentration. For the GPs synthesized through the "microenvironment-imprinting" method, a specific amount of HCl or NaOH was added to the monomer solution. The surfactant, CTAB, was then added to the monomer solution followed by heating to 70 °C. TBAm was dissolved in a small amount of methanol (0.64 g/mL) and injected into the hot monomer solution afterwards. Then, the solution was degased by purging with N_2 while stirring at 70 °C for 1 h. An aqueous solution of AAPD (140 mg/mL) was later added by syringe. The polymerization reaction was carried out at 70 °C for 3 h under a N_2 atmosphere, as illustrated in Scheme 1. The resulting mixture was purified by dialysis against an excess volume of water over four days (water was changed more than three times a day). The anion-exchange of the GPs was conducted to remove trace amounts of counter anions and CO_2 absorbed by the amines during dialysis.

3. Quantification of hydrodynamic diameters, VPTTs, and swelling ratios of GPs

The hydrodynamic diameters and scattering intensities of GPs were measured by dynamic light scattering (DLS) (Zetasizer Nano, Malvern Instruments Ltd.). The GP solutions were sealed within quartz cells filled with N_2. The solutions were equilibrated at each temperature for 6 min prior to the measurement. The relative scattering intensity is defined by equation (1), where I_{max} and I_{min} is the maximum and minimum scattering intensity respectively. The VPTT was determined as the temperature at which the plateau of the relative scattering intensity begins. The swelling ratio is defined as the ratio of the diameters at 30 °C (D_{30}) and 75 °C (D_{75}), as described by equation (2).

$$Relative\ scattering\ intensity = \frac{I - I_{min}}{I_{max} - I_{min}} \qquad (1)$$

$$Swelling\ ratio = \frac{D_{30}}{D_{75}} \qquad (2)$$

4. Quantification of amine content in GPs

A SevenMulti pH meter equipped with a pH probe (InLab® Routine Pro, METTLER TOLEDO) was calibrated with three standard buffers (pH 7.00, 4.01, and 9.21) prior to use. The pH titrations with 0.01 M HCl were performed at 30 °C and 75 °C under N_2 purging with stirring at 1000 rpm. The temperature of the GP solution was controlled using a water bath.

5. Measurement of pK_a shift curve during heating process

Since the apparent pK_a is defined as the pH value at the half-neutralization point, the pK_a shift of the GP solution was measured with the pH probe as follows. The half-neutralized GP solution was prepared by adding 0.5 eq of HCl to the GP solution after quantification of the GP amine content by pH titration. Then, under a N_2 atmosphere with stirring at 1000 rpm, the pH value and temperature of the solution were recorded by the pH meter every 3 s during the heating process. The temperature was controlled by a personal organic synthesizer (EYELA PPW-2000, Tokyo Rikakikai Co., Ltd.).

6. Preparation of GP films

The lyophilized GPs were dissolved in methanol. The hydrogel films were then prepared by casting the methanol solutions containing 120 mg of GPs on the inner bottom surface of a stainless steel container with a surface area of 120 cm^2. After completely evaporating the methanol, 4 mL of water was added per gram of GPs.

7. Measurement of CO_2 absorption capacity of GP films

The CO_2 absorption capacities of the GP films were quantified by a grease-free steel and Teflon tube system. As illustrated in Scheme S1, 10% CO_2 (90% N_2, 10 mL/min) gas was passed through 60 °C water to generate saturated water vapor at that temperature. The resulting 60 °C water-saturated gas mixture was channeled into a stainless steel container with the GP film on the inner surface, and then to a gas chromatograph (GC, Shimadzu Corp.) after condensing the water moisture at 5 °C. The GC was equipped with a thermal conductivity detector (TCD) and a Shincarbon ST 50/80 column (2 m, Shinwa Chemical Industries Ltd.; carrier gas: He, 40 mL/min; column temperature: 210 °C; detector temperature: 240 °C). All the GP films were equilibrated under the gas flow for about 2 h at 30 °C. Thereafter, the CO_2 desorption (at 75 °C) and absorption (at 30 °C) capacities of each film were quantified by monitoring the CO_2 concentration every 1.7 min by the GC-TCD for 25 min and 46 min, respectively.

Scheme S1. Gas flow in the CO_2 absorption/desorption quantification system

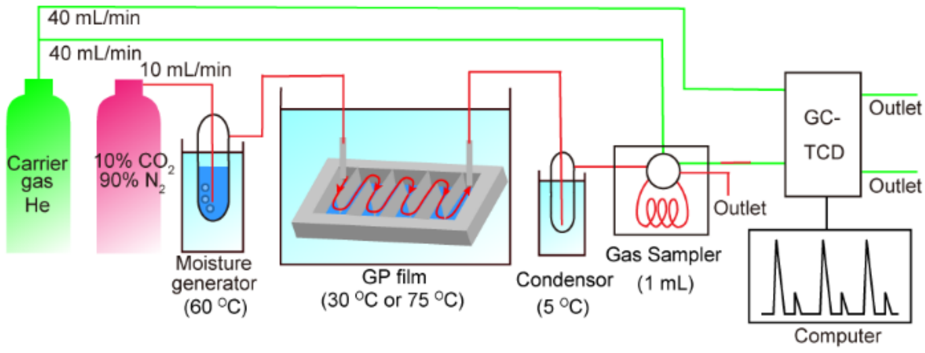

8. Effect of pK_a values of GPs on the reversible CO_2 capture stoichiometry of GP films

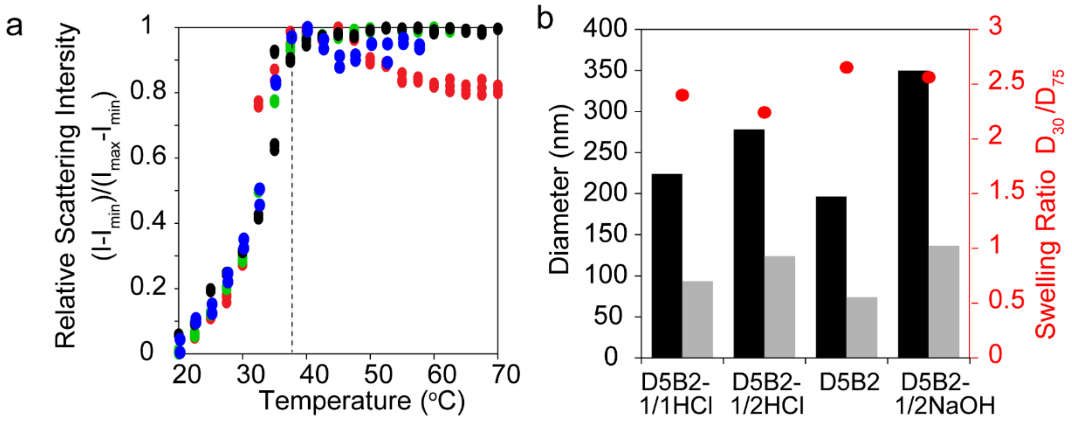

Fig. S1. (a) Relative scattering intensity as a function of temperature for D5B2 (blue), D5B2-1/1HCl (red), D5B2-1/2HCl (green), and D5B2-1/2NaOH (black). (b) Diameters (left Y-axis) at 30 °C (black bar) and 75 °C (gray bar) and swelling ratios (right Y-axis, red dot) between 30 °C and 75 °C of the GPs.

9. Reversibility of GP film of D55B2T43

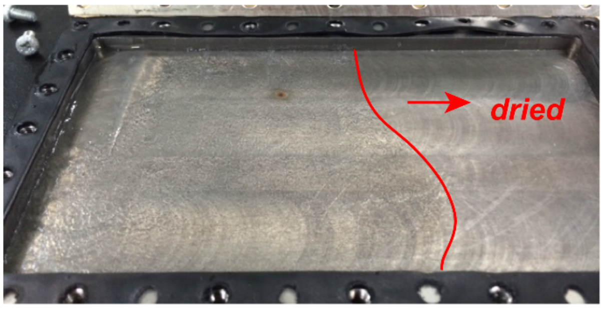

Fig. S2. Photograph of the GP film after the first 10 cycles.
